# Deep learning for electrocardiogram interpretation: Bench to bedside

**DOI:** 10.1111/eci.70002

**Published:** 2025-04-07

**Authors:** Bas B. S. Schots, Camila S. Pizarro, Bauke K. O. Arends, Marish I. F. J. Oerlemans, Dino Ahmetagić, Pim van der Harst, René van Es

**Affiliations:** ^1^ Department of Cardiology University Medical Center Utrecht Utrecht The Netherlands; ^2^ Cordys Analytics B.V. Utrecht The Netherlands

**Keywords:** artificial intelligence, cardiovascular diseases, deep learning, electrocardiogram, implementation

## Abstract

**Background:**

Recent advancements in deep learning (DL), a subset of artificial intelligence, have shown the potential to automate and improve disease recognition, phenotyping and prediction of disease onset and outcomes by analysing various sources of medical data. The electrocardiogram (ECG) is a valuable tool for diagnosing and monitoring cardiovascular conditions.

**Methods:**

The implementation of DL in ECG analysis has been used to detect and predict rhythm abnormalities and conduction abnormalities, ischemic and structural heart diseases, with performance comparable to physicians. However, despite promising development of DL algorithms for automatic ECG analysis, the integration of DL‐based ECG analysis and deployment of medical devices incorporating these algorithms into routine clinical practice remains limited.

**Results:**

This narrative review highlights the applications of DL in 12‐lead ECG analysis. Furthermore, we review randomized controlled trials that assess the clinical effectiveness of these DL tools. Finally, it addresses different key barriers to widespread implementation in clinical practice, including regulatory hurdles, algorithm transparency and data privacy concerns.

**Conclusions:**

By outlining both the progress and the obstacles in this field, this review aims to provide insights into how DL could shape the future of ECG analysis and enhance cardiovascular care in daily clinical practice.

## Graphical abstract

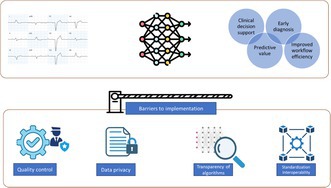

## 1 INTRODUCTION

Since its discovery by the Dutch scientist Willem Einthoven over a century ago, the electrocardiogram (ECG) has become an essential tool in clinical practice. Its operation is based on detecting the electrical signals that propagate through the body when cardiac cells depolarize and repolarize, using electrodes positioned on the body's surface. The ECG has been fundamental in diagnosing arrhythmias, myocardial ischemia and in identifying noncardiac disorders, such as electrolyte imbalances.^1^, ^2^ Due to its low cost, noninvasive nature, and ease of acquisition, this instrument is widely available worldwide. However, detecting ECG changes associated with pathological processes requires a certain level of expertise, particularly when it comes to subtle changes that enable early diagnosis.^3^ Moreover, great variability in ECG analysis and in accuracy of diagnoses have been observed among physicians when compared to experts.^4^

In recent years, the increase in availability of large‐scale datasets combined with the increase in computing power has allowed for the use of artificial intelligence (AI) to become a prominent topic in many different areas of research, including medicine.^5^ Furthermore, FDA regulatory bodies have approved AI‐based products at an increasing rate, with a strong focus on machine learning (ML) and deep learning (DL) technologies.^6^ Where ML methods require manual feature extraction, DL uses neural networks to automatically learn complex features and patterns from large datasets. This is especially promising in the clinical context, where much data is acquired, and expert opinion is not always readily available. Frequently used terms in the AI field are defined in Table 1.

**TABLE 1:** Definitions of key artificial intelligence and deep learning terms used in ECG analysis.

Term	Description
Artificial intelligence (AI)	The concept of computer systems being able to carry out complex tasks mimicking human intelligence.
Machine learning (ML)	A subset of AI in which the model finds patterns in the data requiring manual feature extraction.
Deep learning (DL)	A subset of ML in which neural networks automatically learn features from large datasets without manual feature extraction
Feature	A property or characteristic of the data, for instance a waveform characteristic like the QRS width.
Label	The target variable that the model is predicting, for instance ‘atrial fibrillation’
Deep neural network (DNN)	A type of neural networks that can automatically learn features from large datasets without manual feature extraction
Convolutional neural network (CNN)	A type of DNN in which convolutional layers are used that are especially well designed for pattern recognition
Residual neural network (Res‐Net)	A special type of CNN that uses connections between layers to leverage information flow across deep networks
Recurrent neural network (RNN)	A type of neural network designed for sequential data analysis, where information from previous inputs is used to influence current processing. RNNs are particularly suited for tasks such as time‐series analysis.
Long short‐term memory (LSTM)	A type of RNN specifically designed to handle long‐term dependencies in sequential data. LSTMs are effective for analysing ECG signals, capturing patterns across longer sequences for tasks like arrhythmia detection.
Auto Encoder	A neural network designed for reconstructing the input data from a compressed state, useful in ECG analysis for noise reduction and feature extraction.
Training	Network training is the process of feeding training data into the model to update its parameters and improve its predictions.
Secondary validation	Using a separate dataset often from another source or patient demographic group to validate the model's performance and test its generalization capabilities.
Area under the receiver operating curve (AUROC)	A performance metric that measures the classification performance at different thresholds. What is good and what is bad

DL methods have already made significant contributions in areas such as medical imaging, disease prediction and drug development.^5^ For ECG analysis, it has already been shown that a variety of cardiac diseases can be accurately detected using DL, in some cases even outperforming medical experts.^7^ Also, in situations where specialized expertise is not available, DL‐based automatic ECG analysis could enhance the interpretability and accessibility of ECG data. Additionally, the automatic extraction of features from raw ECG data enables the identification of unknown (subtle) patterns associated with known diseases or patterns linked to pre‐disease stages, that may be imperceptible to the human eye, offering predictive and prognostic value.

**FIGURE 1:**
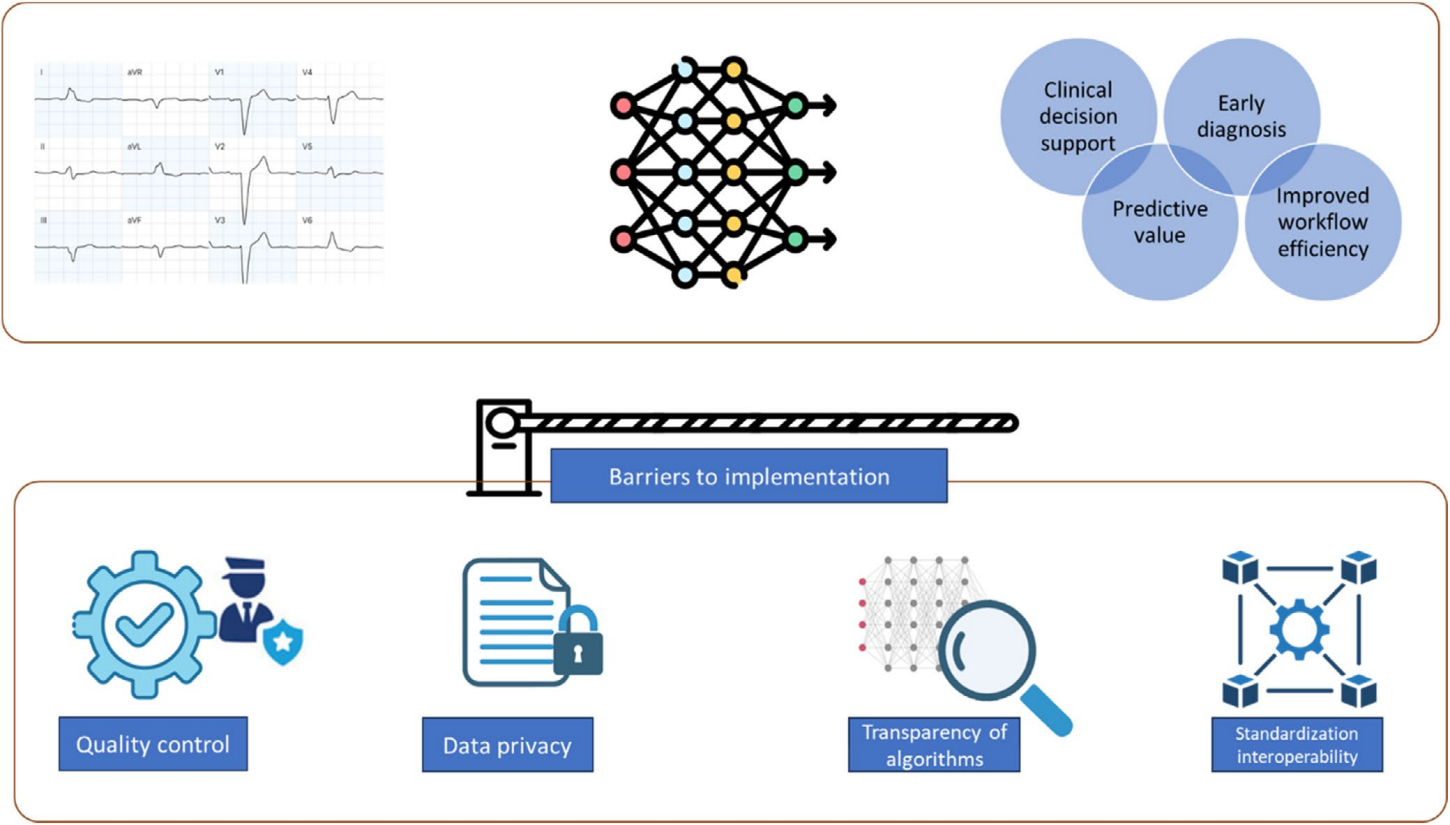
Schematic overview of applications of DL‐enabled ECG analysis and the barriers to implementation of DL tools to clinical practice.

Deep learning for ECG analysis has been reviewed previously, highlighting its suitability for this task and presenting applications in different domains.^8^, ^9^, ^10^ The current review provides an update on DL for ECG analysis and places additional emphasis on the implementation in clinical practice, including regulatory and practical barriers. Given that the 12‐lead ECG is the most common method of ECG acquisition in clinical practice, this review focused exclusively on studies that included more than 10,000 12‐lead ECGs. We first provide an overview of current DL clinical applications. Subsequently, we focus on the implementation of ECG‐based DL models in daily clinical practice and highlight the ongoing challenges to implementation (Figure 1).

### 1.1 Current clinical applications

To explore the applications of DL in ECG analysis, a comprehensive PubMed search was conducted. The articles identified were screened by two independent authors (BS and CP). To highlight the broad applicability of DL ECG analysis, cardiac disorders were grouped into three categories: rhythm and conduction disorders, structural and functional diseases and ischemic heart disease.

## 2 RHYTHM AND CONDUCTION DISORDERS

**FIGURE 2:**
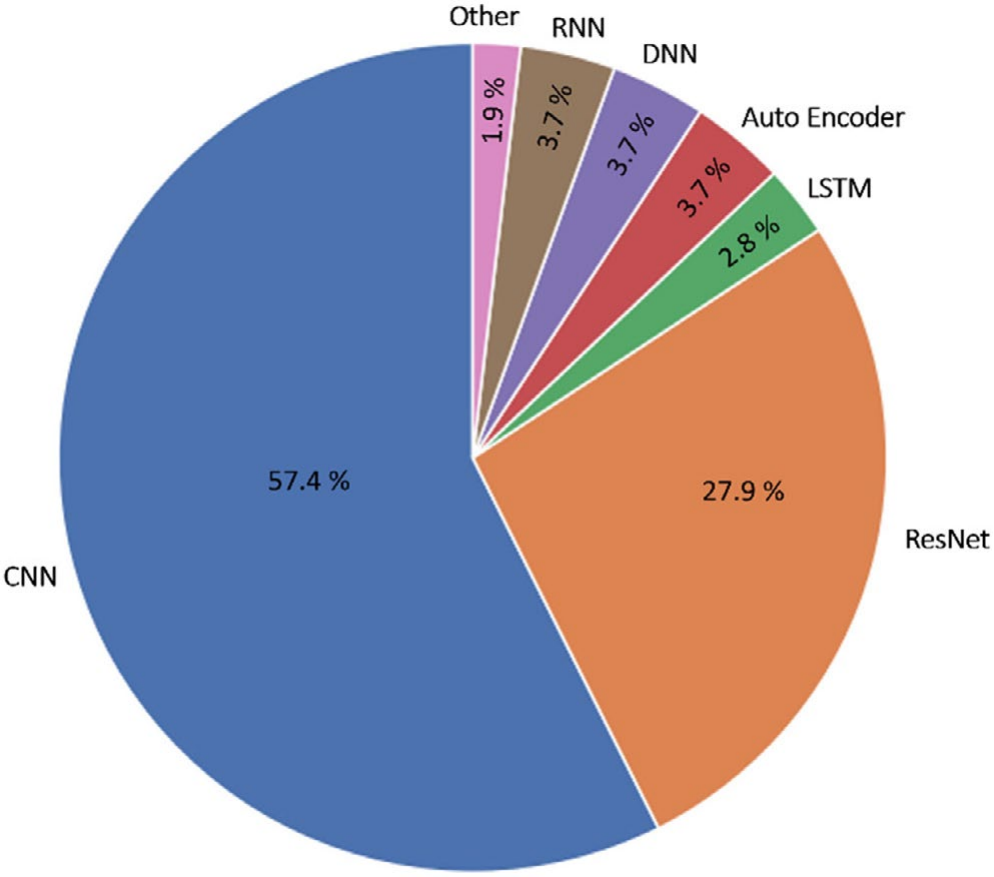
DL architectures used in DL ECG analysis. Overview of model architectures used in DL ECG analysis. CNN, convolutional neural network; DNN, deep neural network; LSTM, long short‐term memory; ResNet, residual network; RNN, recurrent neural network.

Synchronized heart contraction and blood propulsion requires a stable propagation of electrical current through cardiomyocytes. Abnormalities in impulse generation or propagation can lead to rhythm and conduction disorders. Hemodynamic repercussions vary among different arrhythmias and identification of arrhythmias that require prompt treatment is important. However, ECG evaluation in the clinic is time consuming and observer dependent. Automation of this process using AI can therefore be of great importance. Many studies have focused on the automatic classification of rhythm disorders from ECGs using DL.^11^ These models can be applied in an end‐to‐end fashion, directly identifying arrhythmias from the ECG input data. Most approaches adopt a convolutional neural network (CNN), a type of DL network in which convolutional operations are used to extract more complex features from the ECG waveforms. Placing these operations in series (layers) allows the algorithm to use complex features from the ECG waveform to predict specified outcomes. Other architectures have also been explored (Figure 2). Both single and multi‐label classification approaches have shown promise.^11^, ^12^, ^13^, ^14^, ^15^, ^16^, ^17^, ^18^, ^19^, ^20^, ^21^, ^22^, ^23^, ^24^, ^25^ For a deeper exploration of technical DL details in ECG analysis, readers are referred to previous reviews on DL ECG.^8^, ^9^, ^10^

Single‐label, or binary classification targets just one specific abnormality at a time. DL algorithms have been shown to perform well at this task, accurately classifying conditions such as atrial fibrillation (AF), identifying premature ventricular complexes and other arrhythmias with high precision.^11^, ^12^, ^13^, ^14^, ^15^ These models can enhance clinical care by improving diagnostic throughput and reducing observer variability, increasing the efficiency of clinical workflow. In a pivotal study, Attia et al. developed a ResNet‐based CNN to detect paroxysmal AF from sinus rhythm ECGs acquired up to 31 days before diagnosis, achieving an AUROC of 0.87.^16^ This showed how DL can identify intrinsic markers of AF onset from normal ECGs, beyond human capability. Building on this, Raghunath et al. trained a model to predict new‐onset AF within 1 year from sinus rhythm ECGs, achieving an AUROC of 0.85.^17^ This shift from detection to prediction highlights DL's potential to transition from diagnostics to preventive care.

Multi‐label classification refers to models that can predict several rhythm abnormalities from a single ECG that may occur simultaneously. Studies have shown that DL approaches are suitable for this task.^11^, ^18^, ^19^, ^20^, ^21^ For example, Kashou et al. developed a CNN trained on over two million ECGs to classify 66 distinct labels, including 23 rhythm disorders, achieving an AUROC >0.98 across all labels.^22^ Similarly, Ribeiro et al. utilized a ResNet‐based CNN to classify six types of arrhythmias and conduction disorders, performing comparably to medical professionals.^23^ These studies underscore the capacity of DL systems to handle multiple abnormalities simultaneously, assisting clinicians and reducing their workload. Other approaches aim to optimize performance by moving toward lead‐specific prediction, where DL networks are designed with separate CNN branches for each of the 12 leads or using only a subset of the 12 leads as input.^24^, ^25^

Often only a subset of these rhythm abnormalities can be consistently predicted, making the model noncomprehensive. Others have investigated more comprehensive triage systems for rhythms disorders, with the aim of classifying the patients in categories of urgency, rather than predicting the specific pathology.^26^, ^27^, ^28^ Such models may be used to reduce clinical workload by improving the prioritization of ECGs and have also been tested in a prospective implementation setting, proving to be more accurate than the currently employed non‐DL based algorithm.^29^

## 3 STRUCTURAL AND FUNCTIONAL DISEASES

### 3.1 Valvular diseases

The cost‐effectiveness of a screening program with echocardiography for diagnosis of valvular heart disease (VHD) in the general population has not been demonstrated.^30^ DL applications may enable ECG‐based diagnosis of VHD. Several strategies have been developed to detect VHD, with aortic stenosis (AS) and mitral regurgitation (MR) being the most studied diseases.^31^, ^32^, ^33^, ^34^, ^35^ Kwon et al. were among the pioneers in using DL for the detection of significant AS in ECGs, achieving an AUROC of 0.884 in an external validation set.^36^ Comparable results were obtained when ECG‐echocardiogram pairs were used in CNN models for detecting moderate–severe AS, highlighting the potential of ECG as a screening tool for identifying patients at high risk of valvular disease and helping to optimize the use of diagnostic resources in settings where access to echocardiography is limited^35^, ^37^

### 3.2 Hypertrophic cardiomyopathy

Hypertrophic cardiomyopathy (HCM) affects 1 in 200–500 people, with potential outcomes including heart failure and sudden cardiac death.^38^ Therefore, early detection might help reduce the risk of adverse outcomes. Ko et al. developed a CNN model to detect hypertrophic cardiomyopathy (HCM) from the ECG, later validated in an independent cohort. The model achieved a high performance to detect HCM, even in patients with a normal ECG.^39^ Recently, this model was further validated using data from an international cohort of HCM patients and non‐HCM controls, with comparable results.^40^ Interestingly, ECGs were analysed without selection for tracing quality or any pre‐processing, making the results comparable with real‐world ECG acquisition.^40^ Automatic identification of HCM patients from electronic health record data has also been reported.^41^ These models could provide an improvement in screening and early diagnosis for HCM, allowing earlier intervention and better disease management. With increasing options for HCM‐related therapies, such as cardiac myosin inhibitors, earlier diagnosis is increasingly relevant for these patients.^42^, ^43^

### 3.3 Left ventricular dysfunction

Left ventricular dysfunction is an independent predictor of mortality in heart failure patients with reduced (HFrEF) and preserved (HFpEF) ejection fraction.^44^, ^45^ It is currently assessed using imaging techniques, but ECG DL allows detecting both ventricular systolic (LVSD) and ventricular diastolic (LVDD) dysfunction, characteristic markers of HFrEF and HFpEF, respectively.^46^, ^47^, ^48^, ^49^, ^50^, ^51^ Attia et al. demonstrated that an AI‐enabled ECG model could accurately predict LVSD (ejection fraction ≤35%).^52^ Validation using routine ECGs from a broad patient population showed its potential as an effective screening tool for asymptomatic individuals.^53^ Yagi et al. investigated the generalizability of a CNN model designed to detect LVSD from 12‐lead ECGs across multiple institutions. The model performed well on internal data, but showed varying performance on external data, underscoring the need for models to be tested across diverse populations before clinical deployment.^54^ An external validation in a European population confirmed the model's potential in detecting LVSD.^55^ It achieved a high negative predictive value (96%) but a lower positive predictive value (39.9%), indicating that further testing is required in broader, nonhospitalized populations. Importantly, they also explored the model's ability to predict future LVSD in patients with preserved ejection fraction (≥50%), demonstrating moderate success.^55^ This predictive capability highlights the potential of AI‐ECG models not only in current diagnosis but also in identifying patients at risk for future LVSD.

## 4 ISCHEMIC HEART DISEASE

Prompt diagnosis of myocardial infarction (MI) is vital in reducing mortality and morbidity.^56^, ^57^ Using DL to complement ECG assessment of patients with suspected acute coronary syndrome could improve diagnostic accuracy and potentially reduce door‐to‐balloon time.^58^ Chang et al. proposed an algorithm to detect acute ST‐elevation MI (STEMI) and achieved an AUROC of 0.98 in an external test set, outperforming board‐certified physicians and commercial ECG algorithms.^59^ Their model was also effective in prehospital settings, reducing response times for paramedics by quickly identifying STEMI.^58^ However, ST‐segment elevation remains an imperfect surrogate marker for coronary occlusion, with 25%–30% of patients presenting with occlusion on angiography, despite not having ST‐segment elevation.^60^ A DL model by Herman et al. has shown the ability to detect occlusive MI based on more complex ECG patterns.^61^ The model's performance was superior to conventional STEMI criteria and comparable to specialized ECG experts, highlighting the potential for such models to improve patient outcomes by capturing those with silent or atypical presentations. Others have extended this creating DL models to detect MI without ST‐segment elevation (NSTEMI), which showed that AI could identify MI cases even in patients without classical ST‐segment elevation.^62^, ^63^ These studies highlight the broader applicability of DL models, not just in STEMI detection, but in providing diagnostic insights for NSTEMI patients, who often present with ambiguous ECG findings.

## 5 RANDOMIZED CONTROLLED TRIALS

Randomized controlled trials (RCTs) are considered essential in demonstrating the effectiveness of an intervention and are a crucial tool to evaluate implementation of DL tools in daily clinical practice. To date, few RCTs investigating the use of DL algorithms for ECG analysis have been conducted.

Yao et al. aimed to assess the potential of an ECG‐based, DL‐powered clinical decision support tool to enable early diagnosis of LVSD (EF ≤50% within 90 days of ECG acquisition),^64^ using the network developed by Attia.^52^ This RCT was conducted in 120 care teams from 45 clinics and hospitals in the USA. ECGs from 22,641 patients were acquired, with 11,573 in the intervention group and 11,068 in the control group. The intervention group received model‐assisted alerts indicating a high risk of LVSD, while the control group followed routine clinical care. LVSD diagnosis rate in the intervention arm was significantly higher, indicating the added benefit of screening individuals using DL ECG analysis.

Adedinsewo et al. conducted an RCT to evaluate DL‐based cardiomyopathy screening in pregnant and postpartum women in Nigeria, a country with the highest global incidence of peripartum cardiomyopathy.^65^, ^66^ The primary endpoint was the detection of LSVD, defined as ejection fraction <50%. A total of 587 women were assigned to the intervention arm and 616 to the control arm. Patients in the intervention arm received DL‐based cardiomyopathy screening, while the control group received usual care with echocardiography at the physician's discretion. However, no significant difference between the two arms was found.

Lin et al. looked at the ability of their model to predict overall mortality in a multisite RCT involving 39 physicians and 15,965 patients from the emergency and inpatient departments. It was shown that implementation of their algorithm significantly reduced the 90‐day mortality rate from 4.3% to 3.6%. Furthermore, physicians were more likely to escalate clinical care in the case where the DL model flagged the patient as high‐risk for mortality.^67^

In the ARISE study, an ECG‐based supporting tool for reducing door‐to‐balloon time for STEMI patients was tested.^68^ Among the 43,234 patients, 145 were diagnosed with STEMI, including 77 in the intervention group and 68 in the control group; the intervention group demonstrated a significantly shorter door‐to‐balloon time of 82 min compared to 96 min in the control group (*p* = 0.002). They concluded that DL‐assisted ECG triage decreased the ECG‐to‐balloon time for patients presenting to the ED as well as for inpatients.^68^ These results underscore the potential of ECG‐AI triage to enhance STEMI patient treatment.

More RCTs have been proposed and are currently under consideration in different application areas for cardiovascular research, for example, for AF risk assessment and coronary artery disease diagnosis.^69^, ^70^

### 5.1 Barriers to the implementation of DL in clinical practice and ongoing efforts to overcome them

**FIGURE 3:**
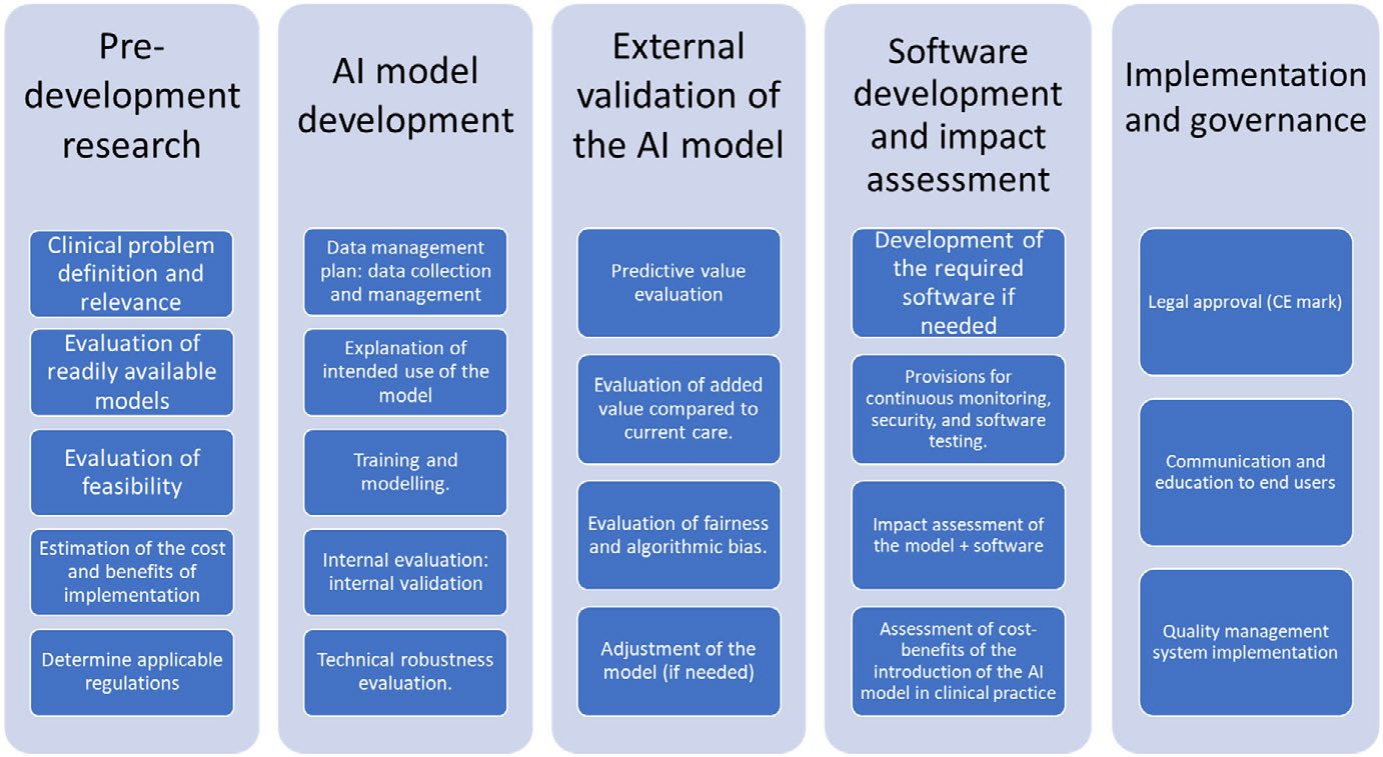
Overview of the steps needed for AI development and implementation.

Integration of DL into daily clinical practice remains an ongoing challenge and is yet to become a widespread reality. Similar to the development of other medical devices, development of DL algorithms needs to follow a structured approach^71^ (Figure 3).

Investments in AI development have surged in recent decades. In 2023, private investment in AI reached €9 billion across Europe. Additionally, the EU, through its Digital Europe Program, is set to invest €7.6 billion to accelerate digital transformation over the next 7 years. This growing interest in AI is further demonstrated by the sharp rise in machine learning publications, including DL, which increased sevenfold between 2015 and 2022.^72^ Similarly, the number of AI‐related medical devices in the cardiovascular field approved by the FDA has risen, from just 5 in 2016 to 60 by 2022.^72^

Moreover, openness from clinicians and patients to integrate AI in daily clinical practice has also been studied. Recently, Arends et al., investigating barriers and facilitators to AI usage in clinical practice, showed that potential end‐users of DL ECG analysis had a positive perception of automatization for ECG analysis and were open to integrating AI in their work [Arends, 2024]. Despite this surge in investment and innovation, implementation of AI developments in healthcare continues to face prominent issues.

## 6 CONCERN FOR PATIENT SAFETY: QUALITY CONTROL OF ALGORITHMS

One barrier to AI implementation is related to the concern of physicians for the accuracy of AI algorithms and how this may affect patient safety. In the study of Arends et al., poor model performance was identified as the most important barrier [Arends, 2024]. Critical evaluation of developed algorithms is crucial for their implementation. Often, external validation is considered an appropriate method to determine models' reliability and generalizability. However, external validation does not guarantee model usefulness.^73^ AI algorithms are considered medical devices; therefore, development and certification of an AI algorithm are regulated and standardized processes. Industry standards are created to provide developers with guidelines to produce compliant and high‐quality products. New standards with basic safety and performance requirements are being developed, applying to all ECG devices, including computerized analysis.^74^ The 80601‐2‐86 standard includes ECG algorithm testing requirements, testing methods and data sets based on the intended used of the algorithm and not the type of ECG device. Furthermore, the use of medical devices in clinical practice is regulated by law. In Europe, CE certification indicated that a medical device complies with the Medical Device Regulation and can be commercialized in the European Economic Union, while in the USA, the FDA oversees regulation. In other regions, regulation follows local laws and standards.^75^, ^76^

In august 2024 the new regulation focused on ensuring safety, transparency and accountability has taken effect in Europe, the European Artificial Intelligence act.^77^ This aims to contribute to the clarification of requirements and obligations for developers and deployers, and it adopts a risk‐based approach. High risk AI systems, including AI‐driven diagnostic tools, medical devices integrated with AI, patient monitoring and risk assessment, must meet strict requirements. These include implementation of risk management frameworks throughout the AI system lifecycle; representative, and bias‐free data for model development; transparency; human oversight; and post marketing monitoring detect any potential harm or safety issues after deployment.^77^ This act marks considerable progress toward enhancing patient safety. However, while it includes provisions to reduce barriers—such as allowing companies to test high‐risk AI applications under regulatory supervision without the full regulatory burden—it may still complicate implementation. Additionally, given the rapidly evolving nature of AI, the current regulations will require continuous revision to keep pace with technological advancements and ensure ongoing relevance and effectiveness.

## 7 DATA SHARING AND PRIVACY

One of the key factors in developing and training AI algorithms is the availability and quality of data. The amount of data needed for training varies based on factors such as the type of learning and the complexity of the model. The amount of data used to develop models ranged from 10,000 to 2.5 million ECGs among studies included in this review. As a result, effective strategies for data sharing, including international collaboration, are crucial to ensure the development and deployment of high‐quality AI tools. However, this raises concerns about patients' rights to data protection and privacy. Since 2018, data sharing within the European Union has been governed by the General Data Protection Regulation (GDPR), which sets strict standards to safeguard individuals' rights and privacy, including limiting data sharing with non‐EU countries.^78^ Despite these protections, some GDPR principles, such as data minimization and the right to erasure, may conflict with AI's reliance on large‐scale datasets, posing challenges to balancing privacy with technological advancement.

Anonymization of data ensures compliance with current EU regulations. However, due to the detailed and granular nature of many datasets, particularly in fields like healthcare, achieving effective anonymization on a large scale is challenging, leaving a significant risk of re‐identification. This risk arises from the possibility that even anonymized data, when combined with other data sources, can inadvertently reveal an individual's identity.^79^ To address these data‐sharing challenges, initiatives such as anonymized benchmarking databases have emerged. These databases provide access to de‐identified datasets while minimizing privacy risks. For example, several international datasets are available for research purposes, including anonymized ECG data (Table 2). Another promising approach to overcoming data‐sharing hurdles is the use of federated learning methods, which allows AI models to be trained across multiple decentralized datasets without directly sharing the data between institutions.^80^ Instead, models are trained locally, and only the learned parameters are shared. This method preserves privacy while enabling the collaborative development of robust AI tools.

**TABLE 2:** Electrocardiography databases.

Database Name	Subjects	No of recordings	No of leads	Duration	Sampling (Hz)	Diagnostic class
MIT‐BIH Arrhythmia Database^88^	47	48	2	30 min	360	Beat annotations (normal beat, LBBB beat, RBBB beat, atrial premature beat, etc) and rhythm annotations (atrial flutter, nodal rhythm, paced rhythm, etc)
PTB Diagnostic ECG Database^92^	294	549	15		1000	MI (148), cardiomyopathy/HF (18), BBB (15), dysrhythmia (14), myocardial hypertrophy (7), valvular heart disease (6), myocarditis (4), miscellaneous (4) and healthy controls (52)2)
PTB‐XL^93^	18,869	21,799	12	10 sec	500	71 classes grouped in five main categories: Normal ECG (9514), MI (5469), ST/T Change (5235), conduction disturbance (4898) and hypertrophy (2649)
Long‐Term ST Database^94^	80	86	2	21–24 h	250	ST segment changes, including ischemic ST episodes, axis‐related non‐ischemic ST episodes, episodes of slow ST level drift and episodes containing mixtures of these phenomena
European ST‐T Database^95^	79	90	2	2 h	250	Changes in ST segment and T‐wave morphology
MIMIC‐III Waveform Database^87^	30,000	67,830	NA	NA	500	Waveforms are unannotated
Lobachevsky University Electrocardiography Database (LUDB)^96^	200	200	12	10 s	500 Hz	Specified heart rate types, position of the electrical axis, specific type of conduction abnormalities, types of extrasystole, types of hypertrophies and ischemia location.
The ECG‐ID Database^97^	90	310	1	20 s	500 Hz	10 annotated beats (unaudited R‐ and T‐wave peaks annotations from an automated detector)
PhysioNet computing in cardiology challenge 2017	NA	8528 (train), 3658 (test)	1	9–60 s	300 Hz	Normal (5154), Atrial fibrillation (771), Other rhythm (2557), Noisy (46)
St. Petersburg Institute of Cardiological Technics 12‐lead Arrhythmia Database	32	74	12	30 min	257 Hz	Acute MI, transient ischemic attack, prior MI, CAD, with hypertension, SV ectopy, AF, SVTA, WPW, AV block, BBB
Shandong Provincial Hospital (SPH) Database^98^	24,666	25,770	12	10–60 s	500 Hz	44 primary classes and 15 modifier, including: Normal ECG, AF, VPCs

Abbreviations: AF, atrial fibrillation; AV, atrioventricular; CAD, coronary artery disease; LBBB, left bundle branch block; MI, myocardial infarction; RBBB, right bundle branch block; SV, supra ventricular; SVTA, supraventricular tachyarrhythmia; VPCs, ventricular premature complexes; WPW, wolf Parkinson white.

## 8 TRANSPARENCY AND MODEL INTERPRETABILITY

Constant development of increasingly complex models raises concerns about their black‐box nature. Transparency in AI has ethical, legal and societal implications and encompasses concepts like explainability, disclosure of the data used for training; and sufficient interpretability to be oversighted by humans.^81^ Several articles included in the GDPR support the concept of ‘right to explanation’, granting individuals the right to meaningful information about the logic involved in automated decision‐making; transparency in processing of personal data; and right to human intervention to challenge or review automatic decisions.^78^ The new European AI act has further specified the obligation that must be followed to comply with existing regulations, particularly for high‐risk systems.^77^ Nevertheless, gaps such as the level of explainability that an AI system must reach or how to ensure human accountability for decisions made by AI remain and continuous reevaluation and specification of regulations may help in the acceptance of integration of AI systems in clinical practice.

Explainable AI provides mechanisms that make the decision‐making process of AI models understandable.^82^ In the context of AI‐enabled interpretation of the ECG, different techniques can be used to identify ECG segments or leads that are the most influential in the model's decision, helping to make the results more transparent and interpretable for clinicians. Post hoc techniques, such as class activation mapping (CAM) and saliency maps can be applied to CNNs to interpret models' prediction. These methods highlight the temporal locations in the ECG that are most relevant to the model's output.^83^, ^84^ However, a key limitation of these techniques is that they do not reveal specific features (e.g. QRS morphology) that influence the prediction. Therefore, their use may be limited, or even lead to various kinds of bias. Furthermore, the validity of such techniques has been questioned.

Recently, Van der Leur et al., introduced a novel approach using a variational auto‐encoder (VAE), to improve explainability of ECG analysis. Unlike post hoc methods, this technique incorporates explainability directly into the model's design.^49^ The VAE was trained to compress the median‐beat 12‐lead ECG into a set of explanatory factors. These factors can subsequently be used in common, interpretable prediction models, such as Cox regression, allowing for the identification of factors associated with the predicted outcome. Using the decoder part of the VAE, the factors associated with specific outcomes can be visualized in a reconstructed ECG, providing insights into what patterns the model has learned. This approach offers explainability both at the individual prediction level and at the model level.^49^

## 9 STANDARDIZATION AND INTEROPERABILITY

Interoperability, particularly regarding Electronic Health Records (EHRs), is essential for deploying AI models in clinical settings. Addressing this issue through consistent ECG data standards and interoperable systems is crucial for ensuring the accuracy, reliability and generalizability of AI‐driven ECG analysis.

Currently, ECG data can be obtained from different devices, including wearables, and in diverse settings, such as ambulatory ECGs, resting ECGs and stress tests. Data standardization enables consistent interpretation across studies. Key techniques for ECG data standardization include signal processing, amplitude and time normalization, and data annotation using standardized formats like MIT‐BIH annotation guidelines.^85^ Format standardization can be achieved using common formats like the European Data Format (EDF), DICOM or Waveform Database format (WFDB). WFDB, an open‐source set of file standards for reading and storing signal data, is widely adopted by both research and clinical applications, notably by PhysioNet databases.^86^, ^87^, ^88^

Another important aspect requiring standardization is the reporting system in AI studies. The lack of uniformity among studies makes comparison and pooled analysis challenging. Adopting standard guidelines for AI data reporting with transparency and completeness is essential for critical assessment and bias identification. Furthermore, it may facilitate development of robust meta‐analyses, which could aggregate data and provide stronger evidence supporting the use of AI tools in clinical practice. In recent years various guidelines to standardize reporting of studies have been developed, from algorithm development and validation (TRIPOD‐AI and STARD‐AI)^89^, ^90^ to large‐scale randomized trials (CONSORT‐AI).^91^

## 10 LIMITATIONS AND CONCLUSIONS

This is not a comprehensive review; the detection and prediction of various other diseases have been investigated using DL but are not included in this work, nor have all papers on specific disease been included due to the shear abundance of available literature.

We can conclude that DL‐enabled ECG analysis is a developing field, with various potential applications in diagnosis and prediction of cardiac and noncardiac diseases. Furthermore, implementation of DL tools in clinical practice is being investigated prospectively in different randomized studies. The results of those studies will help to evaluate the feasibility of implementing these algorithms in real‐world clinical practice and their utility compared to current systems. However, implementation of AI systems into the workflow of healthcare systems remains to face several barriers, including concerns related to accuracy and safety, data protection, transparency of the algorithm and interoperability. Despite advances in regulations further investigation and specific guidelines are needed to ensure an increase in deployment of novel AI developments into clinical practice.

## AUTHOR CONTRIBUTIONS

Bas B.S. Schots and Camila S. Pizarro: Conceived study design, conducted the literature analysis, wrote and revised the manuscript, and approved the final manuscript. Bauke K.O. Arends and Dino Ahmetagić: Contributed to the literature analysis, contributed to manuscript revisions, and approved the final manuscript. Marish I.F.J. Oerlemans and Pim van der Harst: Contributed to manuscript revisions, provided supervision, and approved the final manuscript. René van Es: Assisted with study design, contributed to manuscript revisions, provided supervision and approved the final manuscript.

## CONFLICT OF INTEREST STATEMENT

The authors declare the following potential conflicts of interest: The Department of Cardiology at UMC Utrecht may receive royalties in the future from sales of deep learning ECG algorithms developed by Cordys Analytics, a spin‐off company. Additionally, René van Es is the Chief Scientific Officer (CSO) and a shareholder of Cordys Analytics. These affiliations and potential financial interests have been disclosed and are being managed in accordance with institutional policies.

## Data Availability

Data sharing is not applicable to this article as no new data were created or analysed in this study.
